# Effect of Structure Hierarchy for Superhydrophobic Polymer Surfaces Studied by Droplet Evaporation

**DOI:** 10.3390/nano8100831

**Published:** 2018-10-13

**Authors:** Nastasia Okulova, Peter Johansen, Lars Christensen, Rafael Taboryski

**Affiliations:** 1 Danapak Flexibles A/S, DK-4200 Slagelse, Denmark; nasok@nanotech.dtu.dk (N.O.); PJO@danapakflex.com (P.J.); LC@danapakflex.com (L.C.); 2 DTU Nanotech, Technical University of Denmark, DK-2800 Lyngby, Denmark

**Keywords:** hierarchical structures, super-hydrophobic surfaces, droplet evaporation, Cassie-Baxter, contact angle hysteresis

## Abstract

Super-hydrophobic natural surfaces usually have multiple levels of structure hierarchy. Here, we report on the effect of surface structure hierarchy for droplet evaporation. The two-level hierarchical structures studied comprise micro-pillars superimposed with nanograss. The surface design is fully scalable as structures used in this study are replicated in polypropylene by a fast roll-to-roll extrusion coating method, which allows effective thermoforming of the surface structures on flexible substrates. As one of the main results, we show that the hierarchical structures can withstand pinning of sessile droplets and remain super-hydrophobic for a longer time than their non-hierarchical counterparts. The effect is documented by recording the water contact angles of sessile droplets during their evaporation from the surfaces. The surface morphology is mapped by atomic force microscopy (AFM) and used together with the theory of Miwa et al. to estimate the degree of water impregnation into the surface structures. Finally, the different behavior during the droplet evaporation is discussed in the light of the obtained water impregnation levels.

## 1. Introduction

The wetting properties of materials are strongly influenced by their surface roughness. Sparked by advancements in scanning electron microscopy (SEM) that led to a resolution of the surface structure of the lotus flower [1], different artificial patterned surfaces have been fabricated in an attempt to mimic this and other bionic effects [2]. Typical lotus-like structures rely on roughness defined on multiple length scales and a hydrophobic surface chemistry. While in nature such structures can comprise up to six levels of hierarchy [3], the current study comprises a two-level hierarchical structure which is compared to the two structure types it is built from. 

A common practical definition of a super-hydrophobic surface includes two requirements: the apparent contact angle (CA) with water has to be above 150°, at the same time the CA hysteresis (the difference between the advancing and receding contact angles) has to be low (below ~10°). As pointed out in several studies, this definition is somewhat arbitrary [4,5], and a more well-defined and more forgiving, yet less practical, definition would be a surface with a water CA exceeding the one for the theoretically most hydrophobic flat surface comprising the closest hexagonally packed -CF_3_ groups having a water contact angle of ~119° [6]. Super-hydrophobicity should, however, not be confused with self-cleaning, as even though the requirements for the CA and CA hysteresis are met, the surface can lose the water-repellent qualities over time and undergo a so-called wetting transition [7,8,9,10]. Such wetting transitions are typically studied through applied pressure [8,11], evaporation [12,13], and vibration of the droplet [14] or using bouncing droplets [15,16]. In this article we study how droplets behave over time when left to evaporate from different surfaces. This approach has been widely used for the investigation of the droplet evaporation rates [17,18] and for analyzing superhydrophobic and patterned surfaces [19,20,21], and several modeling techniques have been proposed to describe this process [19,22,23,24]. Erbil published a review on the topic in 2012 [25].

The apparent contact angles measured on rough surfaces are conventionally described in terms of the so-called Wenzel [26] and Cassie-Baxter [27] models from 1936 and 1944, respectively. The models are based on a thermodynamic approach whereby the Gibbs free energy is minimized in terms of the CA for the system comprising the three states of matter, namely, the solid substrate, the liquid droplet, and the surrounding gas [28]. Although, a prediction of the apparent CAs based on this approach often fails, and has led to heated scientific debates about its validity [29,30,31], it is conceptually well established that a droplet in the so-called “Wenzel state” completely wets the surface texture, while a droplet in the so-called “Cassie-Baxter state” rests on the summits of the surface texture. The shortcomings of the Wenzel and Cassie Baxter models seem to be associated with droplet pinning, where droplets get trapped in metastable states representing local minima in the free energy [16]. Stated in terms of force arguments, pinning may also stem from minute elastic deformations due to the vertical projection of a Young-type reaction force per unit length of the triple line [32]. Pinning effects are also considered responsible for the so-called contact angle hysteresis [33,34]. For pointy surface protrusions having typical opening angles 2α, it can be argued, by imposing the requirement of the CA on a microscale being equal to the Young CA, $\theta_{Y}$, that the droplet will end up in the Wenzel state if $\theta_{Y}<\alpha +90{}^{\circ}$, while for larger Young CA, the triple phase line will move and impregnate the texture until $\theta_{Y}=\alpha +90{}^{\circ}$ is fulfilled corresponding to a partly wetted surface texture [35]. A more realistic Cassie-Baxter type of equation to predict the apparent CA, θ, for a partially wetted surface was given by Miwa et al. [36]. Here, we state this model in terms of the impregnation depth, Z, measured from the summits of the protrusions, such that positive Z values are obtained when water impregnates the surface texture from above.

(1) $$\;\cos\theta =R_{f}\cdot \varphi \left(Z\right)\cdot \cos\theta_{Y}+\varphi \left(Z\right)-1\;\;$$

where $R_{f}>1$ is the Wenzel roughness parameter, i.e., the actual surface area to the projected surface area, $\theta_{Y}$ is the Young contact angle, and φ(Z) is the ratio of the projected wetted area to the total area. The problem with Equation (1) is, however, that the parameter φ(Z) is usually not known. Hence, in this study, we employ AFM measurements to determine the range of half opening angles α(Z) of structures at a given impregnation depth, Z, and impose the condition,

(2) $$\;\theta_{Y}=\alpha \left(Z\right)+90{}^{\circ}\;\;\;\;$$

to estimate Z. When used together with φ(Z), also determined by atomic force microscopy (AFM), we are able to predict the apparent CAs by using Equation (1) and compare them with experiments. Thus, one of the novel findings in this work is a proposed method to estimate the level of wetting on the micro-level and to exploit this information to enable a theoretical computation of contact angle data based on actual measured surface shapes.

Measurements of the rough surfaces in Si and other hard materials are important for understanding the wetting properties, however, for real-life applications the structures need to be transferred to a cheaper materials platform allowing for mass-production of large areas. For this study, the initial Si structures are replicated in polypropylene (PP) flexible foils via a roll-to-roll extrusion coating method (R2R EC), which is a well-established method in the packaging industry. Extrusion coating has a capacity for the manufacturing of up to 2 m wide packaging foils at the production speed up to 1000 m/min. Production of micro- and nano-patterns using R2R EC is a relatively new method that has shown promising results for scaling up the production of biomimetic surfaces [37,38,39].

## 2. Materials and Methods 

Fabrication of the examined surfaces is divided into the following steps: production of the master structure in Si, transfer of the structure into a mold, and subsequent thermoforming of the structures using the R2R EC process [39]. A soft mold (Inmold Flexible Stamp, Inmold A/S, Hørsholm, Denmark) was used for replication of the structures by thermoforming in polypropylene (PP) by R2R EC.

**Figure 1:**
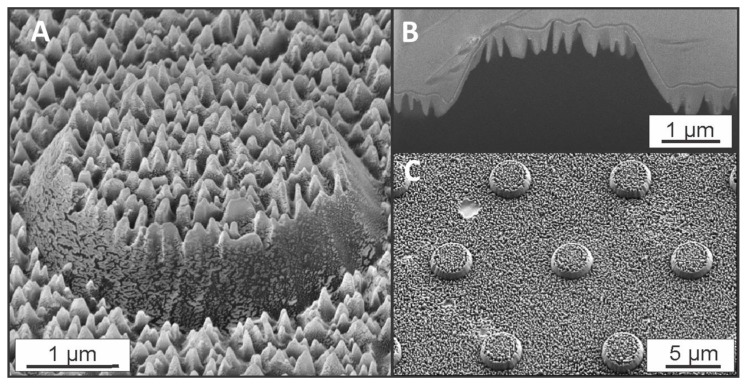
A hierarchical pattern produced in polypropylene (PP) using a roll-to-roll extrusion coating process. (**A**) The pattern coated with 10 nm Au at 45° angle. (**B**) Focused ion beam-SEM (FIB-SEM) cross-section of the pattern, image taken at 52° tilting angle, using tilt compensation function. (**C**) An overview image of the pattern in PP (with 10 nm Au), taken at 30° angle.

The master Si structures were produced using a combination of ultraviolet UV lithography and a deep reactive ion etching step (DRIE) [8]. In total, three types of structures were investigated: micro-sized pillars, needle-like random pattern (referred to as nano-grass), and a combination of the two (the hierarchical structures). SEM images of the produced hierarchical structures are presented in Figure 1.

The photolithography method for producing the micro-pillars is summarized here. The 100 mm Si n-type wafers were spin-coated with positive tone resist AZ5214E (SSE Spinner, Maximus 804, Chemnitz, Germany), patterned using a UV-mask-aligner with a mask of the hexagonally arranged circular pattern (SUSS Mask Aligner MA6, Garching, Germany), and developed for 60 s (AZ 351B developer, Wiesbaden, Germany). Then, the patterns were etched by DRIE using a Bosch process (creating a straight side-wall with 150 nm scallops) (Pegasus DRIE, STS. MP0636, Surface Technology Systems plc, Newport, UK). The excess photoresist was removed directly in the DRIE machine by the oxygen plasma ashing.

The nano-grass pattern was produced using a recipe described in detail by Schneider et al. [35]. In summary, the patterns were formed on 100 mm Si wafers using etching in the DRIE machine (Pegasus DRIE, STS. MP0636, Surface Technology Systems plc, Newport, UK). The needle-like pattern was achieved by alternation of corrosive and passivating gasses, SF_6_/CH_4_ and O_2_, respectively. For this study, the SF_6_/CH_4_ flow rate of 70 sccm and O_2_ flow rate of 90 sccm were used. The produced pattern is commonly known as “black silicon” due to its black appearance on the wafer achieved by the anti-reflection effect of the surface texture.

The hierarchical patterns were obtained by UV lithography and consecutive pattern transfer of the micro-pillars as described above, including the resist removal, followed by the nano-grass formation. The two processes were done directly after each other without removing the sample out of the chamber. The positive relief Si master templates were then sent to InMold A/S for relief inversion and fabrication of soft molds.

The replication using R2R EC was done as described in previous publications [37,39], but in short: soft molds were attached to the cooling roller of a pilot R2R EC machine (Danapak Flexibles, Slagelse, Denmark). The structured layer consisted of polypropylene (WF420HMS, Borealis AG, Vienna, Austria) with a density of 0.9 g/cc. The process was performed at constant force and constant extruder output at 10 m/min line-speed, and cooling roller temperature 70 °C. PP was laminated onto a 36 µm thick polyethylene terephthalate (PET) carrier foil. 

Foils were cut into manageable pieces (5 cm × 5 cm) and characterized by scanning electron microscopy (SEM), (SEM Zeiss Supra 40 VP, Oberkochen, Germany) after sputter coating with a ~10 nm thin gold layer. Focused ion beam SEM (FIB-SEM), (FEI Helios EBS3, ThermoFisher Scientific, Oregon, USA) was done after deposition of a conformal platinum protection layer on the structures. The sample morphology was obtained by atomic force microscopy (AFM), (NX20, Park Systems, Suwon, Korea) using a tapping mode AFM probe (Tap300DLC, budgetsensors). AFM data taken from (5 µm × 5 µm) scan areas were analyzed using SPIP 6.2.2 software (Image Metrology A/S, Hørsholm, Denmark) and MATLAB (MathWorks, Inc., Massachusetts, USA).

Contact angles where measured with a tilting cradle tensiometer fitted with a high speed camera (Attension Theta optical tensiometer, Biolin Scientific AB, Gothenburg, Sweden). Prior to each measurement, the surface charge was neutralized with ionized air (Zerostat 3, Mility, Sigma-Aldrich Denmark A/S, Copenhagen, Denmark). The tensiometer cradle was tilted at 1°/s while capturing images of drop profiles with 1 frame per second. For each measurement, a 6.5 µL droplet was placed on the surface. The static contact angle for the sessile droplet was measured using the Young-Laplace fit. The stage was then tilted and the contact angle hysteresis was recorded just before the roll-off (for this value, a polynomial fit on the bottom half of the droplet was used, as the Young-Laplace fit fails to find a solution for a misshaped droplet during the stage tilt) [40]. For each nanostructured foil, five recordings were made with water drops in different areas.

For the evaporation recordings, each droplet started at ~6.5 µL and was left evaporating for 30 minutes in ambient atmosphere. The droplet shape was recorded every second, and the apparent contact angle was fitted for each frame using the Young-Laplace fit. The values extracted from the fit are the apparent contact angle on both sides of the droplet and the droplet volume (calculated using the measured cross-section area, given that the static droplet is rotationally symmetric). Each measurement was repeated twice at the same place.

## 3. Results and Discussion

In this study, three types of surfaces are compared: micro-patterned pillar surfaces (as presented by Okulova et al. [39]), randomly patterned nano-grass surfaces (as investigated by Telecka et al. [38]), and hierarchical surfaces, where the micro-pillars are superimposed with the nano-grass structures. A micrograph of the hierarchical pattern is presented in Figure 1. The preliminary study on morphologies of the nano-grass has been conducted by Schneider et al. [35] and an optimized structure is used in this study.

### 3.1. Contact Angle and Droplet Evaporation

The results of the measurements are presented in Table 1. It is worth noticing that according to these measurements, both the hierarchical structures and the nano-grass samples are superhydrophobic and have very similar wetting properties.

**Figure 2:**
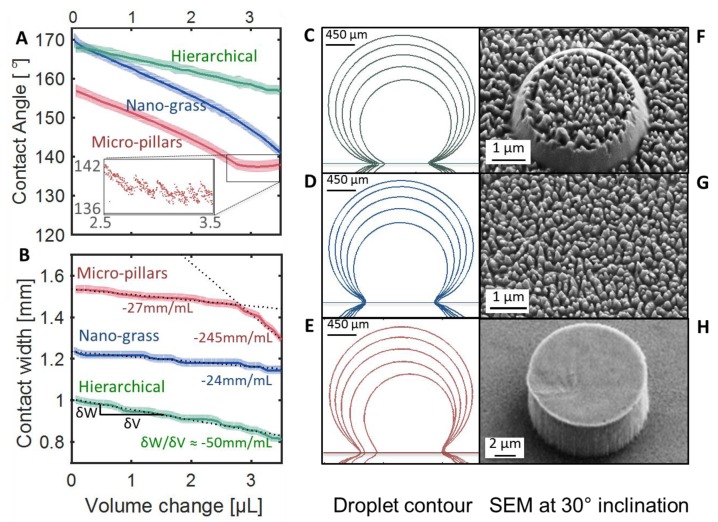
Results of the droplet evaporation experiments. A water droplet left on the PP surface for 30 min. (**A**) The water contact angle compared to the droplet evaporated volume for the three types of structures. Insert: zoomed in view of a part of the curve for the micro-pillar surface. (**B**) The contact diameter a) with respect to the evaporated volume V. Each line is fitted with a linear fit. The fits are shown as black dotted lines. The slope values from the fits are written under each curve. (**C**), (**D**), (**E**) The contours of one droplet sitting on each of the three surfaces during the evaporation. (**F**), (**G**), (**H**) SEM micrographs of the surface structures for hierarchical, nano-grass, and micro-pillar samples, respectively. The images are taken at a 30° tilt angle, and each sample is coated with 10 nm Au for better imaging.

The surfaces are further characterized by CA recordings for sessile droplets resting on the structured surfaces during evaporation. The contact angles for each type of structure during evaporation are shown in Figure 2A, plotted as a function of the evaporated volume from each sessile droplet. Each curve is an average of two independent measurements (the shown standard deviation is calculated for the average of the two measurements and angles on both sides of the droplet).

The data shows an interesting effect of the hierarchy: the apparent CA for the pure nano-grass sample decreases much faster than for the hierarchical sample. The two samples had seemingly the same CA and CA hysteresis properties to start with, however, the difference between the two structures is evident after the 30 min evaporation. The hierarchical surfaces seem to have a similar rate of contact angle decrease as the micro-pillar surfaces; an attempt to explain this behavior is presented in the last part of this article. Another noticeable effect is a change in the behavior for the micro-pillar sample. The second part of the curve has a slope around one, which could be due to the droplet reaching the receding contact angle and hence jumping from one pillar to another (a zoom-in on the area is presented in the insert of Figure 2A).

In order further to analyze the measured effect, contact diameter values are extracted from each frame using MATLAB. The contact diameter here is the diameter of the circle enclosed by the triple line—where air, water, and PP are in contact. The results of these measurements are presented in Figure 2B. The contours of the droplet during evaporation are presented in Figure 2C–E, and the corresponding structure micrographs are seen in Figure 2F–H. The stair-like shape of the contact line plot comes from the limit of the resolution of the camera; each step simply corresponds to the pixel-size. Each curve is fitted with a linear fit and the slope of each curve is shown directly under each plot. 

**Figure 3:**
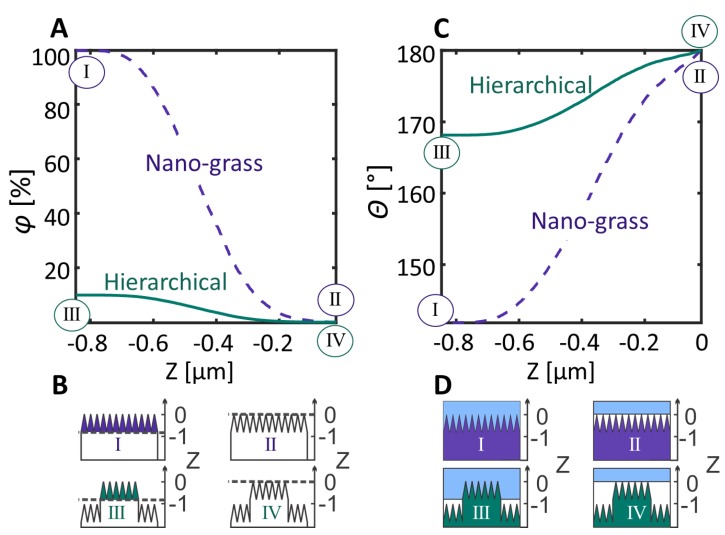
The estimation of the wetting by using the modified Cassie-Baxter equation. (**A**) The filling factor φ (projection of the wetted area/total area) as a function of the wetting depth Z (note, the zero-value is set to the top of the pattern); the values are obtained from the atomic force microscopy (AFM) measurements of the structures. (**B**) A schematic of the wetting depth at the points marked in the graph. (**C**) The expected contact angle at different wetting depths, Z (zero value is zero at the top here as well). (**D**) A schematic of the process in C.

The contact angle measurements seem to be in agreement with the contact diameter measurements. The contact line shrinks twice as fast for the hierarchical structures than for the nano-grass structures. This is also seen in the contour lines in Figure 2D, the droplet is more pinned to the nano-grass than to the other two structure types shown in Figure 3C,E. Confirming the results from the contact angle measurements, the micro-pillar sample reaches the receding contact angle and seems to start unpinning faster, and the contact diameter drops more rapidly. The contours of the droplets seem to be more pinned on one side than the other, which is not surprising when taking the possible defects on the nano-pillar surface and the stochastic nature of the nanograss structure into account. The droplet on the hierarchical surface in Figure 3C seems, however, mainly to evaporate in constant CA mode, whereas the droplet on the plain nano-grass surface in Figure 3D mainly seems to evaporate when in constant contact diameter mode as described by Kulinich and Farzaneh [24]. This hints that the droplet sitting on the hierarchical surface is less pinned than the droplet on the plain nano-grass surface. The last droplet in Figure 3E, is clearly strongly pinned in an asymmetric mode.

The micro-pillar pattern is at this point assumed to be in Cassie-Baxter state at all times, the contact angle for the presented micro-pillar pattern calculated using Wenzel equation [26] is 104.5°, while the Cassie-Baxter contact angle [27] for the same structure is 157.0°, which is in agreement with the results presented in Table 1 for the apparent CA.

The produced micro-pillar structures were designed to never undergo a wetting transition from the Cassie-Baxter state into the Wenzel regime. According to Jung and Bhushan [41], the full wetting transition will occur for similar pillar-patterns at pitches above 50 µm. The wetting transition for the current pattern will take place only when the droplet volume decreases below 10–20 µm, and at this size the contact angle is not detectable for the used equipment (the lowest droplet size used in this study was ~700 µm).

### 3.2. AFM Measurements and the Wetting Level

The nano-grass covered samples were imaged using AFM and the data from the measurements were used for the estimation of the wetting level. First, the supposedly wetted area of the pattern was used for calculating the φ(Z) (the projected area over the full area of the sample), here, the integral of the structure height distribution (Abbott curve) was used. This value was then used directly in Equation (1) to calculate the expected contact angle at each depth of the pattern (note that in this calculation the tips of the black silicon structure were set as the zero value). The results of this calculation are presented in Figure 3.

For these calculations, the roughness parameter $R_{f}$ was read out from the AFM measurements and was taken as a constant average for all the surfaces with nano-grass. The value used in all calculations was $R_{f}$ = 3.78. The apparent contact angle of a water droplet on a flat PP surface (a replica of the part of the shim based on a polished Si wafer) was used as the value for the Young contact angle, $\theta_{Y}\;$= (102 ± 1)°.

**Figure 4:**
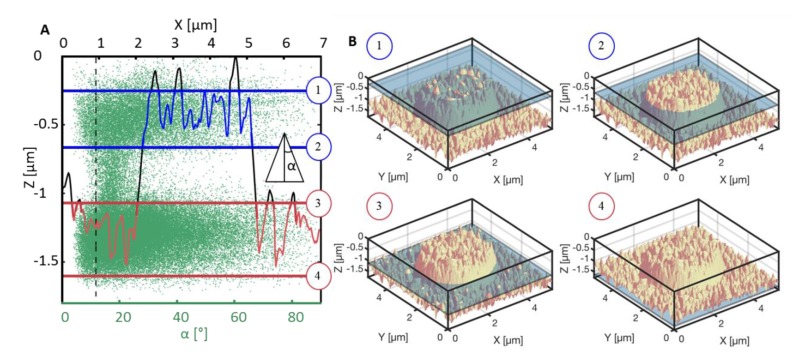
(**A)** The half-opening angles as the function of the depth into the pattern. The green dots represent the distribution of all half-opening angles α across the AFM measured hierarchical surface (the bottom horizontal axis). The black line shows one one-dimensional (1D) cut through the middle of the pillar pattern (the top horizontal axis). The blue lines highlight the top part of the nano-grass pattern, where the half-opening angle is below 12°, where the wetting should not be able to take place. The red lines show the bottom part of the nano-grass pattern, where the opening angles are below 12°, which is another place where further wetting should not be possible. The three-dimensional (3D) representation of the mentioned critical areas of the pattern marked in blue and red are shown in (**B**) 1–2 and 3–4 respectively. Compared to the results in Table 1 and Figure 3, the wetting level should correspond to the situation in B2, where the water has wetted the top layer of the nano-grass pattern but does not yet touch the nano-grass carpet between the pillars.

The surface topography obtained from AFM measurements was used for calculating the half-opening angle $\alpha \left(Z\right)=acot\left(\sqrt{{\left(dZ/dX\right)}^{2}+{\left(dZ/dY\right)}^{2}}\right)$, where dZ/dX and dZ/dY values were extracted directly from the AFM data. The measured values for all (X,Y) coordinates are plotted against the Z-measured height in Figure 4A. In order to visualize where on the pattern the particular opening angle is found, a one-dimensional (1D) cut through the dataset through the middle of the pillar is shown as the black line. The critical α(Z) value was calculated using Equation (2) and for PP foils with apparent contact angle on the flat surface of 102°, used here as the Young contact angle, α(Z) = 12°. This critical angle is shown in the graph with a vertical dashed black line. For all the values below this angle, the wetting should not be possible according to Equation (2).

Two areas, where a significant amount of opening angle values are below 12° can be distinguished from the graph: on the top part of the pillar, where the top layer of the nano-grass pattern is shown (blue) and the valley area, where the second layer of the nano-grass pattern is present (red). Both areas are framed with horizontal lines, and for each of the 4 Z-values, a 3D AFM image including the expected wetting degree is sketched out in Figure 4B (1–4, respective to each line in the Figure 4A). Compared to the FIB-SEM image of the structure (Figure 1B), the AFM tip experiences a slight tip convolution, which creates many faulty high values of the opening angle at every top and bottom part of the grass pattern. Due to the random nature of the nano-grass pattern, the height of each individual tip varies, which makes it difficult to trust an average value of the opening angle. However, the values for each middle part of the needles must be close to the true value, and hence can be trusted.

To summarize the results in Figure 4, the level of wetting in such a nano-grass covered sample must lie somewhere between the blue lines or between the red lines, as that is where the local water-air-substrate interaction prevents the water from travelling further down along the protrusion. Compared to the results from the calculated theoretical value of the contact angle according to the Miwa model, originated from the modified Cassie-Baxter equation) presented in Figure 3, the contact angle for the hierarchical pattern should lie between Z-values of −0.7 µm and −0.25 µm, which corresponds to the contact angle range 168°–176°. If compared to the measured value of the contact angle for the hierarchical pattern, if this theory is correct, the wetting of the hierarchical pattern must correspond to Figure 4B (2), where the top part of the pillar is covered with water but does not touch the bottom part of the nano-grass carpet. This could explain why the hierarchical structure experiences less pinning: the water droplet is only in contact with 1/10 of this nano-grass pattern for these structures compared to the pure nano-grass sample. For the hierarchical structures, the unpinning from each pillar-top seems to have a lower threshold than unpinning from the fully covered nano-grass “carpet”.

## 4. Conclusions

In this study, the strong effect of a two-level hierarchical structure on prolonged life of hydrophobicity of a polymer sample is shown through droplet evaporation. An attempt to explain this effect showed a possible wetting level for a nano-rough surface compared to a hierarchical micro/nano-rough surface. A method for estimation of the wetting level using direct results from the AFM measurements is presented.

In further studies, different types of more robust nano-patterns should be tested, as well as a larger number of hierarchy levels. The mass-production roll-to-roll platform seems to give robust reproducible results, and the scalability of the pattern production, including techniques to allow for larger origination areas, should be investigated. 

## Figures and Tables

**Table 1:** The wetting properties of the different structure types: the static contact angle (CA), the contact angle hysteresis while tilting the surface, and a photo of the water droplet resting on the structured surface.

Structure	Hierarchical	Nano-Grass	Micro-Pillars
**Contact angle**	167° ± 0.5°	170° ± 2°	157° ± 1°
**CA hysteresis**	6° ± 2°	8° ± 1.5°	16° ± 2.5°
**Static droplet**	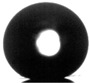	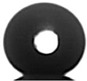	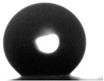

**Figure A1:**
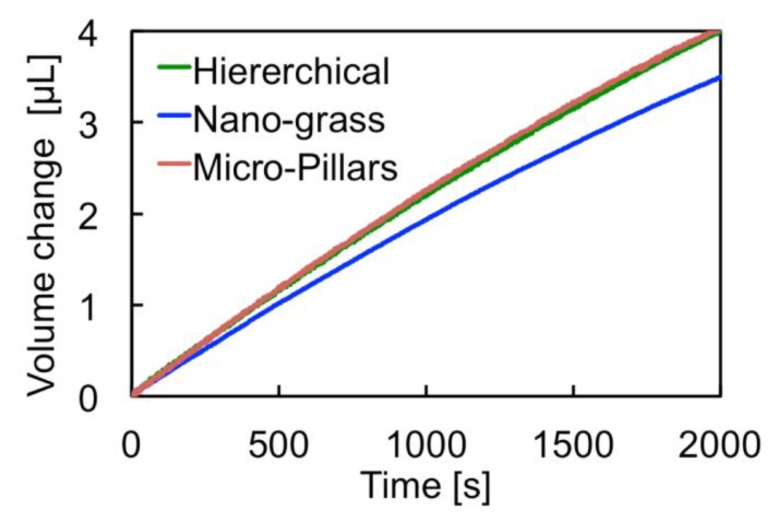
The droplet volume change (evaporated volume) with respect to time. The volume of the droplet pinned on a nano-grass surface decreases slower than that of the droplet resting on the structures with micro-pillars.

## Appendix A

The droplet evaporation was recorded over time and the results presented in the article refer to the contact angle, the contact diameter, and the volume of the droplet. The volume of the droplet, however, changed over time for different structure types. In Figure A1 the evaporation rate is shown. The droplet seems to evaporate faster on micro-pillar and hierarchical structured samples than on the nano-grass samples. This effect could originate in the fact that samples patterned with micro-pillars have a larger surface area exposed to air [42].
